# Genetic polymorphisms of *PGF* and *TNFAIP2* genes related to cervical cancer risk among Uygur females from China

**DOI:** 10.1186/s12881-020-01144-5

**Published:** 2020-10-27

**Authors:** Zumurelaiti Ainiwaer, Reyilanmu Maisaidi, Jing Liu, Lili Han, Sulaiya Husaiyin, Jing Lu, Mayinuer Niyazi

**Affiliations:** Department of Gynecology, Xinjiang Medical University, People’s Hospital of Xinjiang Uygur Autonomous Region, No 91 Tianqi Road, Urumqi, Xinjiang, 830001 China

**Keywords:** *PGF*, *TNFAIP2*, Polymorphism, Cervical cancer

## Abstract

**Background:**

*PGF* and *TNFAIP2* are important angiogenic factors, which were abnormal expression in cervical cancer (CC). However, there is currently no report investigating the relationship of *PGF* and *TNFAIP2* gene polymorphisms to CC risk.

**Methods:**

We conducted a case-control study of 342 CC patients and 498 cancer-free controls in a Chinese Uygur female population. Three SNPs (*PGF* rs8019391, *PGF* rs2268615, and *TNFAIP2* rs710100) were selected and genotyped to assess the possible association of *PGF* and *TNFAIP2* polymorphisms with CC susceptibility. Logistic regression analysis adjusted by age was used.

**Results:**

*PGF* rs2268615 (OR = 1.39, 95% CI = 1.04–1.86, *p* = 0.024) and *TNFAIP2* rs710100 (OR = 1.44, 95% CI =1.07–1.95, *p* = 0.018) polymorphisms were associated with the increased risk of CC. Moreover, T allele of *PGF* rs8019391 was highly represented in patients with stage III–IV compared with stage I-II (OR = 2.17, *p* = 4.58 × 10^− 4^). MDR analysis revealed a positive interaction between the SNPs.

**Conclusion:**

Our data indicated that *PGF* rs2268615, and *TNFAIP2* rs710100 polymorphisms might be risk factors for CC susceptibility, which contributed to the increased risk of CC.

**Trial registration:**

Not applicable.

**Supplementary information:**

**Supplementary information** accompanies this paper at 10.1186/s12881-020-01144-5.

## Background

Worldwide, cervical cancer (CC) is the fourth most common cancer, with an estimated 570,000 cases and 311,000 deaths in 2018 [1]. Cervical cancer is the most common cancer of female genital system in China and the incidence of CC tends to be younger [2]. A wide range of inter-individual genetic variability and other pathogeneses might contribute to cervical carcinogenesis. Accumulating evidence suggested that single nucleotide polymorphisms (SNPs) in tumor-associated genes played important role in the genetic susceptibility to CC, such as FAS, IL-17A, IL-17F and HOTAIR polymorphisms [3–5].

*PGF* (placental growth factor) gene, also named *PLGF*, encodes a homologous of vascular endothelial growth factor. *PGF* has been reported as a potent stimulator in cancer invasion by activating angiogenesis [6]. In addition, the overexpression of *PGF* is correlated with tumor stage, cancer progression and metastasis [7]. *TNFAIP2* (TNF alpha induced protein 2) is a primary response gene of TNFα, and the expression of TNFAIP2 is regulated by various transcription factors and signaling pathways, including NF-κB, KLF5 and retinoic acid pathways [8]. *TNFAIP2* is an important angiogenic factor, and is significantly associated with intratumoral microvessel density [9]. In addition, previous studies reported the abnormal expression of *PGF* and *TNFAIP2* in human cancer, including CC [6, 10]. However, there is no report about the associations between *PGF* and *TNFAIP2* polymorphisms and CC risk. We hypothesized that genetic variants in *PGF* and *TNFAIP2* could contribute to CC susceptibility.

Here, *PGF* rs8019391 and rs2268615, and *TNFAIP2* rs710100 were selected as candidate SNPs from the NCBI dbSNP database (http://www.ncbi.nlm.nih.gov/projects/SNP) and the 1000 Genomes Project data (http://www.internationalgenome.org/), based on minor allele frequency (MAF) of at least 5% in Chinese populations, with a pairwise r^2^ > 0.80 and the call rate of genotyping > 95%. The aim of our study was to investigate the possible association between three SNPs (*PGF* rs8019391, *PGF* rs2268615, and *TNFAIP2* rs710100) and the risk of CC in Chinese Uygur female population.

## Subjects and methods

### Study participants

This study protocol was approved by the Ethics Committee of the People’s Hospital of Xinjiang Uygur Autonomous Region and was in accordance with the guidelines of the Declaration of Helsinki. Written informed consents were obtained from all participants.

Three hundred fourty-two cervical cancer patients (cases, 43.27 ± 11.78 years) and 498 age-matched cancer-free individuals (controls, 43.46 ± 13.03 years) were enrolled from the People’s Hospital of Xinjiang Uygur Autonomous Region, as shown in Table 1. All recruited subjects were unrelated ethnic Han Chinese females. Patients were newly diagnosed and histopathologically confirmed primary cervical cancer according to the clinical staging standards of the International Federation of Gynecology and Obstetrics (FIGO). Among patients, 132 cases were stage I–II, 80 cases were stage III–IV and 130 cases were missing. Patients with any history of other cancers, radiotherapy, chemotherapy, or surgery and inflammatory diseases were excluded. The controls were recruited from the health checkup in the same hospital during the same period. Controls had no any history of cancers and diseases of the liver, kidneys, heart, brain, and vascular system, as well as gynecological inflammatory diseases. Demographic characteristics and clinical information were collected from the standardized questionnaires.

**Table 1:** Characteristics of patients with cervical cancer and controls

Characteristics	Cases (***n*** = 342)	Controls (***n*** = 498)	***p***
**Age**			
Mean ± SD (years)	43.27 ± 11.78	43.46 ± 13.03	0.832
> 43	176 (51.5%)	263 (52.8%)	
≤ 43	166 (48.5%)	235 (47.2%)	
**HPV status**			
Negative	51 (14.9%)		
Positive	195 (57.0%)		
Missing	96 (28.1%)		
**Stage**			
I-II	132 (38.6%)		
III-IV	80 (23.4%)		
Missing	130 (38.0%)		

### SNP selection and genotyping

Peripheral venous blood samples (5 mL) were obtained from all subjects and stored in EDTA-coated tubes. Genomic DNA was extracted using the GoldMag DNA Purification Kit (GoldMag Co. Ltd., Xi′an City, China) according to the manufacturer’s protocol, then quantified by NanoDrop 2000 (Thermo Scientifc, Waltham, MA, USA), and stored at − 20°Cfor further experiments. *PGF* rs8019391 and rs2268615, and *TNFAIP2* rs710100 were selected as candidate from the NCBI dbSNP database (http://www.ncbi.nlm.nih.gov/projects/SNP) and the 1000 Genomes Project data (http://www.internationalgenome.org/), based on minor allele frequency (MAF) of at least 5% in Chinese populations, with a pairwise r^2^ > 0.80, and the call rate of genotyping > 95%. SNPs. miRNASNP_v2 database (http://bioinfo.life.hust.edu.cn/ miRNASNP2/index.php) and HaploReg v4.1 (https://pubs.broadinstitute.org/mammals/haploreg/ haploreg.php) were used to predict the potential function of these polymorphisms (Supplementary Table 1). These candidate SNPs were genotyped with Agena MassARRAY system (Agena, San Diego, CA, U.S.A.) as described previously [11, 12], and performed by two laboratory technicians in a double-blinded fashion. The primers for PCR amplification and single base extension were designed using the Assay Design 3.0 software (Supplementary Table 2). For quality control, approximately 10% of the samples were randomly selected and repeated genotyping, and 100% concordance rate was observed.

### Data analysis

All statistical analysis was performed using SPSS version 18.0 (SPSS Inc., Chicago, IL, USA) and PLINK software. A student’s t-test was performed to analyze the differences in the age distribution between patients and controls. The Hardy–Weinberg equilibrium (HWE) was tested by a goodness-of-fit χ^2^ test for each SNP among the control subjects. The genotype and allele frequencies between two groups were compared usingχ^2^ test. The association of candidate SNPs with CC risk was assessed by odds ratios (ORs) and 95% confidence intervals (CIs) using logistic regression analysis for both combined and respective genotype [13]. Stratification analysis by demographic and clinic variables was also performed to assess the genetic association. Multifactor dimensionality reduction (MDR) (version 3.0.2) was used to evaluate the SNP–SNP interactions in CC risk [14]. A two-tailed *p*-value < 0.05 was considered to be statistically significant for all the analyses.

## Results

The results of genotyping was shown in Additional file 2. In Supplementary Table 3, the MAF of *PGF* rs8019391 and rs2268615, and *TNFAIP2* rs710100 between the case and control groups were listed. The genotype distribution of these SNPs in controls were in accordance with the Hardy-Weinberg equilibrium (*p* > 0.05). The call rate for rs8019391, rs2268615 and rs710100 were 100, 99.7 and 99.1%, respectively. The MAFs distribution of *PGF* rs2268615-A allele and *TNFAIP2* rs710100-A allele were higher in the case group, which increased the risk of CC (rs2268615, A vs C, OR = 1.27, 95% CI = 1.03–1.58, *p* = 0.029; and rs710100, A vs G, OR = 1.23, 95% CI = 1.01–1.50, *p* = 0.043).

The results of multiple genetic model adjusted by age revealed *PGF* rs2268615 and *TNFAIP2* rs710100 conferred to the increased CC risk (Table 2). *PGF* rs2268615 was associated with an increased risk of CC under heterozygote (OR = 1.39, 95% CI = 1.04–1.86, *p* = 0.024), dominant (OR = 1.40, 95% CI = 1.06–1.84, *p* = 0.018) and log-additive (OR = 1.29, 95% CI =1.03–1.61, *p* = 0.027) models. For rs710100 in *TNFAIP2*, compared with GG genotype, GA genotype (OR = 1.44, 95% CI =1.07–1.95, *p* = 0.018) and GA + AA genotype (OR = 1.42, 95% CI = 1.07–1.89, *p* = 0.016) increased 1.44-fold and 1.42-fold CC risk, respectively. Moreover, the result of the additive model also showed an increased risk of CC (rs710100, OR = 1.23, 95% CI = 1.00–1.50, *p* = 0.046). However, there was no significant association between *PGF* rs8019391 and CC susceptibility.

**Table 2:** Relationships between the candidate SNPs and cervical cancer risk

Gene SNP ID	Model	Genotype	Case	Control	Adjusted by age and gender	
					OR (95%CI)	***p***
PGF rs8019391	Genotype	CC	208	327	1.00	
		CT	119	145	1.29 (0.96–1.74)	0.093
		TT	15	26	0.91 (0.47–1.76)	0.777
	Dominant	CC	208	327	1.00	0.150
		CT-TT	134	171	1.23 (0.93–1.64)	
	Recessive	CC-CT	327	472	1.00	0.585
		TT	15	26	0.83 (0.43–1.6)	
	Log-additive	–	–	–	1.13 (0.89–1.42)	0.324
PGF rs2268615	Genotype	CC	160	273	1.00	
		CA	156	191	**1.39 (1.04–1.86)**	**0.024**
		AA	26	31	1.43 (0.82–2.49)	0.209
	Dominant	CC	160	273	1.00	**0.018**
		CA-AA	182	222	**1.40 (1.06–1.84)**	
	Recessive	CC-CA	316	464	1.00	0.453
		AA	26	31	1.23 (0.72–2.11)	
	Log-additive	–	–	–	**1.29 (1.03–1.61)**	**0.027**
TNFAIP2 rs710100	Genotype	GG	118	210	1.00	
		GA	171	211	**1.44 (1.07–1.95)**	**0.018**
		AA	53	69	1.37 (0.89–2.08)	0.150
	Dominant	GG	118	210	1.00	**0.016**
		GA-AA	224	280	**1.42 (1.07–1.89)**	
	Recessive	GG-GA	289	421	1.00	0.576
		AA	53	69	1.12 (0.76–1.65)	
	Log-additive	–	–	–	**1.23 (1.00–1.50)**	**0.046**

*SNP* single nucleotide polymorphism, *OR* odds ratio; *95% CI* 95% confidence interval

*p* values were calculated by logistic regression analysis with adjustments for age and gender

*p* < 0.05 means the data is statistically significant

Age stratification displayed that *PGF* rs2268615 and *TNFAIP2* rs710100 increased the risk of CC among women at age ≤ 43 years (Table 3). After calculating the ORs for the allele (OR = 1.38, *p* = 0.041 and OR = 1.42, *p* = 0.018, respectively), genotype (CA vs CC, OR = 1.55, *p* = 0.039; and AA vs GG, OR = 1.97, *p* = 0.031, respectively), dominant (OR = 1.56, *p* = 0.030; and OR = 1.57, 95%, *p* = 0.034, respectively), and log-additive (OR = 1.40, *p* = 0.042; and OR = 1.42, *p* = 0.020) genetic models, they all displayed the genetic association of *PGF* rs2268615 and *TNFAIP2* rs710100 with CC susceptibility.

**Table 3:** Relationships between the candidate SNPs and cervical cancer risk according to the stratification by age

SNP ID	Model	Genotype	> 43 years				≤ 43 years			
			Case	Control	OR (95%CI)	*p*	Case	Control	OR (95%CI)	*p*
PGF rs2268615	Allele	C	253	393	1.00	0.303	223	344	1.00	**0.041**
		A	99	131	1.17 (0.87–1.59)		109	122	**1.38 (1.01–1.88)**	
	Genotype	CC	89	147	1.00		71	126	1.00	
		CA	75	99	1.25 (0.84–1.86)	0.282	81	92	**1.55 (1.02–2.36)**	**0.039**
		AA	12	16	1.22 (0.55–2.70)	0.626	14	15	1.62 (0.74–3.56)	0.228
	Dominant	CC	89	147	1.00	0.269	71	126	1.00	**0.030**
		CA-AA	87	115	1.24 (0.85–1.82)		95	107	**1.56 (1.05–2.33)**	
	Recessive	CC-CA	164	246	1.00	0.795	152	218	1.00	0.481
		AA	12	16	1.11 (0.51–2.41)		14	15	1.31 (0.61–2.81)	
	Log-additive	–	–	–	1.17 (0.86–1.60)	0.317	–	–	**1.40 (1.01–1.92)**	**0.042**
TNFAIP2 rs710100	Allele	G	217	331	1.00	0.597	190	300	1.00	**0.018**
		A	135	191	1.08 (0.82–1.43)		142	158	**1.42 (1.06–1.90)**	
	Genotype	GG	64	111	1.00		54	99	1.00	
		GA	89	109	1.41 (0.93–2.14)	0.105	82	102	1.46 (0.94–2.27)	0.092
		AA	23	41	0.96 (0.53–1.74)	0.890	30	28	**1.97 (1.07–3.63)**	**0.031**
	Dominant	GG	64	111	1.00	0.208	54	99	1.00	**0.034**
		GA-AA	112	150	1.29 (0.87–1.91)		112	130	**1.57 (1.03–2.38)**	
	Recessive	GG-GA	153	220	1.00	0.418	136	201	1.00	0.103
		AA	23	41	0.80 (0.46–1.38)		30	28	1.59 (0.91–2.79)	
	Log-additive	–	–	–	1.07 (0.81–1.41)	0.635	–	–	**1.42 (1.06–1.90)**	**0.020**

*SNP* single nucleotide polymorphism, *OR* odds ratio, *95% CI* 95% confidence interval

*p* values were calculated by logistic regression analysis with adjustments for age

*p* < 0.05 indicates statistical significance

Subsequently, stratification analysis by tumor stage showed that the risk effect for *PGF* rs8019391 appeared to be more prominent in the subset of patients with stage III + IV (Table 4). Compared with the C allele, rs8019391 T allele was highly represented in patients with stage III–IV as compared to patients with stage I–II under the allele (OR = 2.17, *p* = 4.58 × 10^− 4^), heterozygote (OR = 2.34, *p* = 0.005), homozygote (OR = 5.76, *p* = 0.015), dominant (OR = 2.59, *p* = 0.001), recessive (OR = 4.13, *p* = 0.045), and log-additive models (OR = 2.36, *p <* 0.001).

**Table 4:** Relationship of clinical stage with *PGF* rs8019391 polymorphism in cervical cancer patients adjusted by age

SNP ID	Model	Genotype	I-II	III-IV	OR (95%CI)	***p***
rs8019391	Allele	C	110	220	1.00	**4.58 × 10**^**−4**^
		T	50	44	**2.27 (1.43–3.62)**	
	Codominant	CC	37	91	1.00	
		CT	36	38	**2.34 (1.29–4.25)**	**0.005**
		TT	7	3	**5.76 (1.41–23.52)**	**0.015**
	Dominant	CC	37	91	1.00	**0.001**
		CT-TT	43	41	**2.59 (1.46–4.60)**	
	Recessive	CC-CT	73	129	1.00	**0.045**
		TT	7	3	**4.13 (1.04–16.45)**	
	Log-additive	–	–	–	**2.36 (1.45–3.86)**	**< 0.001**

*SNP* single nucleotide polymorphism, *OR* odds ratio, *95% CI* 95% confidence interval

*p* values were calculated by logistic regression analysis with adjustments for age

*p* < 0.05 indicates statistical significance

**Fig. 1:**
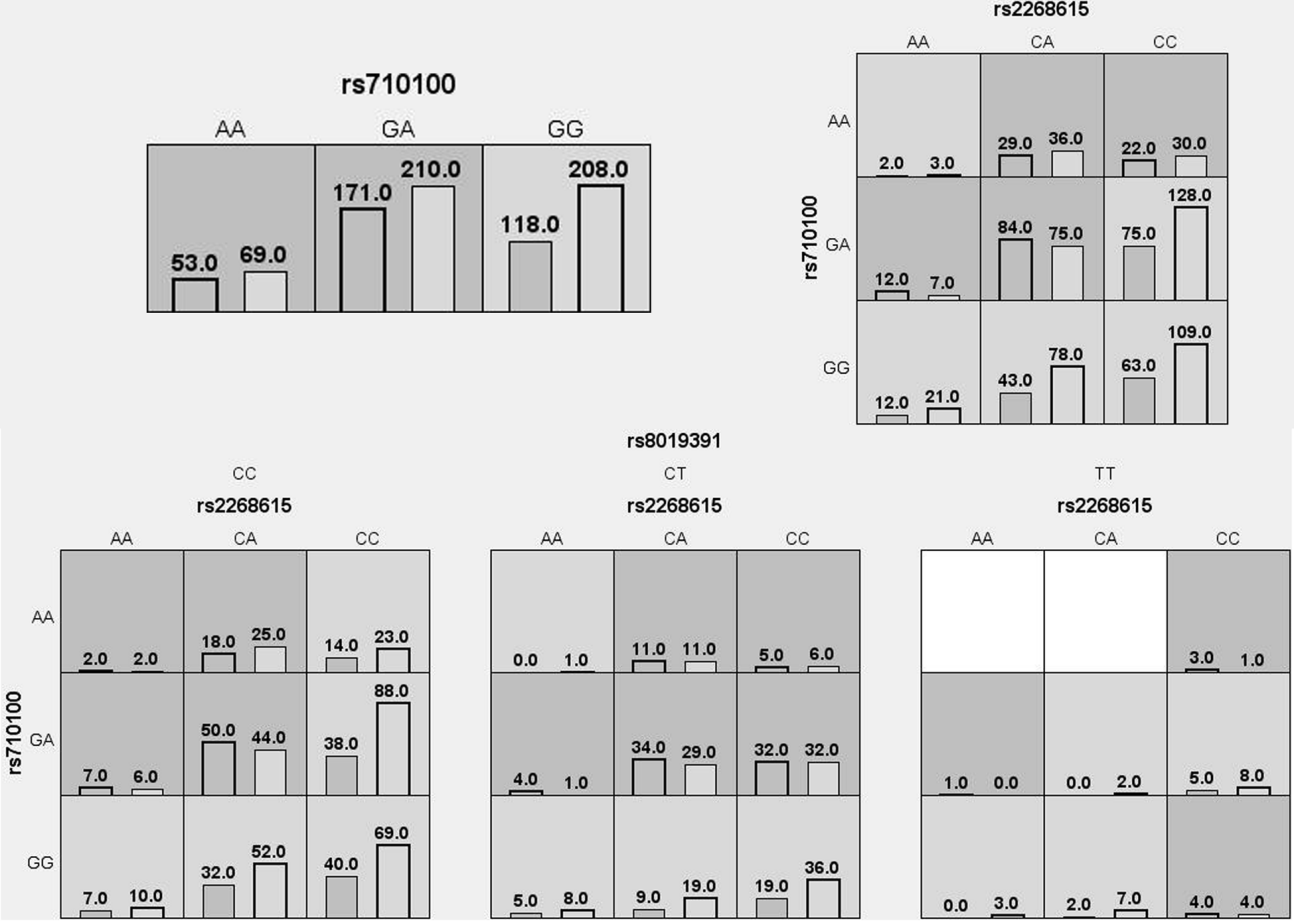
Summary of MDR gene-gene interaction. Each cell shows counts of “case” on left and “control” on right

**Fig. 2:**
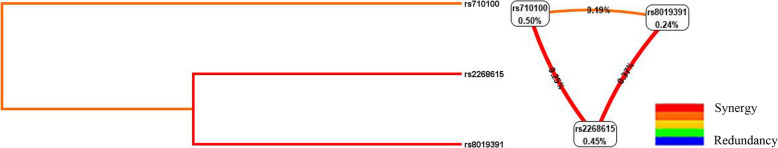
SNP-SNP interaction dendrogram and Fruchterman-Reingold

Subsequently, MDR analysis was implemented to assess the impact of SNP-SNP interaction. Association of higher order interaction with CC risk was summarized in Fig. 1. The result revealed the additive effect between *TNFAIP2* rs710100-GA, *PGF* rs2268615-CA, and *PGF* rs8019391-CT on conferring risk towards the susceptibility to CC. The result of dendrogram and the Fruchterman-Reingold (Fig. 2) showed that *PGF* rs2268615, *TNFAIP2* rs710100, *PGF* rs8019391 exhibited a strong synergy effect on CC risk. Table 5 showed that *TNFAIP2* rs710100 was the best single-locus model to predict the risk of CC (testing accuracy = 0.508, CVC = 6/10, *p* = 0.014). The best two-locus model was the combination of *PGF* rs2268615 and *TNFAIP2* rs710100 (testing accuracy = 0.536, CVC = 9/10, *p* < 0.0001. The three-locus model included *TNFAIP2* rs710100, *PGF* rs2268615, and *PGF* rs8019391 (testing accuracy = 0.550, CVC = 10/10, *p* < 0.0001).

**Table 5:** SNP–SNP interaction models of the *PGF* and *TNFAIP2* genes analyzed by the MDR method

Model	Training Bal. Acc.	Testing Bal. Acc.	CVC	OR (95% CI)	***p***
*TNFAIP2* rs710100	0.544	0.508	6/10	1.46 (1.08–1.97)	**0.014**
*PGF* rs2268615, *TNFAIP2* rs710100	0.564	0.536	9/10	1.91 (1.41–2.59)	**< 0.0001**
*PGF* rs2268615, *TNFAIP2* rs710100, *PGF* rs8019391	0.587	0.550	10/10	2.11 (1.56–2.84)	**< 0.0001**

*MDR* multifactor dimensionality reduction, *Bal. Acc*. balanced accuracy, *CVC* cross–validation consistency, *OR* odds ratio, *CI* confidence interval

*p* values were calculated using χ^2^ tests

*p* < 0.05 indicates statistical significance

## Discussion

In this case–control study, we assessed the association between three SNPs (*PGF* rs8019391, *PGF* rs2268615, and *TNFAIP2* rs710100) and CC susceptibility in the Chinese Uygur female population. We found that *PGF* rs2268615, and *TNFAIP2* rs710100 were associated with the increased risk of CC. Our findings also suggested some possible interaction between genetic variations (*PGF* rs2268615 and *TNFAIP2* rs710100) and age in CC risk. Moreover, the risk effect for *PGF* rs8019391 appeared to be more prominent in patients with stage III + IV. To the best of our knowledge, this is the first report describing that *PGF* and *TNFAIP2* polymorphisms might be risk factor for CC.

*PGF*, located at 14q24.3, belongs to the vascular endothelial growth factor family and presents on various cell types. The expression of *PGF* in tissue or plasma of cancer patients was upregulated in most human tumor types, including gallbladder, gastric and prostate cancers. *PGF* regulates certain cellular processes such as survival, vascular endothelial cell growth, invasiveness, and *PGF* is also involved in pathological angiogenesis and metastasis [15–17]. Previous researches indicated that *PGF* was overexpressed in CC tissues, serum and vaginal lavage compared with adjacent normal tissues or normal women group [6, 18]. *PGF* promotes migration by regulating the expression of epithelial-mesenchymal transition-related protein in CC [6]. These evidence led us to propose the hypothesis that *PGF* could be of importance in CC occurrence. In our study, we firstly assess the association of *PGF* polymorphisms (rs8019391 and rs2268615) with CC susceptibility, and found that rs2268615 conferred the increased risk of CC, and rs8019391 was a risk factor for patients with stage III-IV. A retrospective population-based study showed that 5-year relative survival rates of CC were 90.9, 71.0, 41.7, and 7.8% for the stage I, II, III, and IV, respectively [19]. Therefore, it is highly speculated that *PGF* rs8019391 polymorphism may affect CC progression. Further large-scale studies are needed to verify our findings.

*TNFAIP2* gene (also named B94) is located on chromosome 14q32, and encodes TNFα-inducible protein 2. *TNFAIP2* participates in the NFκB and KLF5 signaling pathway to regulate cell inflammatory, angiogenesis, cell proliferation, migration and invasion [20, 21]. The expression of *TNFAIP2* was found to be abnormal in various cancers, including breast cancer, esophageal squamous cell carcinoma, and glioma [20, 22, 23]. The expression of *TNFAIP2* was significantly increased in CC tissues compared with normal tissues based on TCGA (The Cancer Genome Atlas) database [8]. In addition, the disruption of *TNFAIP2* cytokine/retinoic acid-inducible gene through viral integration contributed to the rapid progression of CC [10]. These evidence suggested that TNFAIP2 might play an important role in the progression of CC. In this study, we found that *TNFAIP2* rs710100 was associated with an increased risk of CC. Rs710100 in the 3′UTR of *TNFAIP2*, located at the predicted miRNAs-binding sites, was related to an increased risk of CC. Specifically, rs1064607 putatively affects the binding of miR-155 [24], whose abnormal expression in CC was correlated with FIGO stage, lymph nodes metastasis, and vascular invasion [25]. Therefore, we proposed that rs710100 in 3′UTR of *TNFAIP2* might affect *TNFAIP2* expression in CC by disturbing mRNA stability or miRNA binding activity, thus causing a higher risk of CC. However, it should be confirmed in further functional studies.

In spite of interesting findings on the association of *PGF* and *TNFAIP2* polymorphisms with CC risk, several limitations need to be addressed. First, there may be selection and information bias since the retrospective study was designed as a hospital-based case–control study. Second, due to insufficient data of HPV screening results and lifestyle data (e.g., smoking), we could not evaluate HPV infection and lifestyle as the potential confounder in the risk estimation of CC.

## Conclusion

In conclusion, our findings indicated a relationship between *PGF* rs2268615, and *TNFAIP2* rs710100 and the increased CC susceptibility in the Chinese Uygur females. Considering that this is the first report on the association of *PGF* and *TNFAIP2* polymorphisms with CC risk, well-designed large and prospective studies are required to validate our findings.

## Supplementary information

**Additional file 1: Supplementary Table 1.** Predicted stability, duplex formation, and functional effect of 3′-UTR polymorphisms in miRNA-binding sites of selected genes. **Supplementary Table 2.** Primers sequence of PCR and UEP used in this study. **Supplementary Table 3.** The information and HWE about the candidate SNPs.

**Additional file 2.**

## Data Availability

The datasets generated and/or analysed during the current study are available in the Additional file 2.

## Abbreviations

CC: Cervical cancer
SNPs: Single nucleotide polymorphisms
*PGF*: Placental growth factor
*TNFAIP2*: TNF alpha induced protein 2
