# Supplementary material for: Genetic polymorphisms of PGF and TNFAIP2 genes related to cervical cancer risk among Uygur females from China
Source: BMC Med Genet. 2020 Oct 27;21:212. doi: 10.1186/s12881-020-01144-5 (PMC7590450; doi:10.1186/s12881-020-01144-5)
Supplement: Supplementary file 1 — Additional file 1: Supplementary Table 1. Predicted stability, duplex formation, and functional effect of 3′-UTR polymorphisms in miRNA-binding sites of selected genes. Supplementary Table 2. Primers sequence of PCR and UEP used in this study. Supplementary Table 3. The information and HWE about the candidate SNPs. [file 12881_2020_1144_MOESM1_ESM.docx]

**Supplementary Table 1. Predicted stability, duplex formation, and functional effect of 3′-UTR polymorphisms in miRNA-binding sites of selected genes**

| **Gene** | **SNP ID** | **Allele** | **Predicted miRNA** | **△ (kcal/mol)** | **miRNA/SNP target duplexes** | **Effect** |
| --- | --- | --- | --- | --- | --- | --- |
| TNFAIP2 | rs710100 | G | [hsa-miR-155](http://www.mirbase.org/cgi-bin/query.pl?terms=hsa-miR-155-5p) | -18.00 | \| 3' \| u \| g \| g \| g \| g \| a \| U \| A \| G \| U \| G \| C \| U \| A \| A \| U \| C \| G \| U \| A \| A \| U \| u \| 5′ \| \| --- \| --- \| --- \| --- \| --- \| --- \| --- \| --- \| --- \| --- \| --- \| --- \| --- \| --- \| --- \| --- \| --- \| --- \| --- \| --- \| --- \| --- \| --- \| --- \| --- \| \|  \|  \|  \|  \|  \|  \|  \| \| \|  \| \| \| \| \| \| \|  \| : \|  \|  \| \| \| \| \| \| \| **\|** \| \| \| \| \| \| \|  \|  \| \| 5' \| g \| c \| t \| g \| t \| g \| A \| G \| C \| A \| C \| C \| G \| C \| C \| A \| G \| C \| [**A**](http://www.ncbi.nlm.nih.gov/projects/SNP/snp_ref.cgi?rs=rs710100) \| T \| T \| A \| g \| 3′ \| | Gain |
|  |  | A |  | -18.40 |  |  |

SNP: single nucleotide polymorphism. Predictions made with the [miRNASNP_v2](file:///C:\Users\slinnell\AppData\Local\Temp\miRNASNP_v2) database: <http://bioinfo.life.hust.edu.cn/miRNASNP2/index.php>

Supplementary **Table 2. Primers sequence of PCR and UEP used in this study**

| **Gene** | **SNP** | **First Primer(5'-3')** | **Second Primer (5'-3')** | **UEP_DIR** | **UEP SEQ (5'-3')** |
| --- | --- | --- | --- | --- | --- |
| PGF | rs8019391 | ACGTTGGATGAAACACTTACCCATGTGCCC | ACGTTGGATGCACAAGAATAATCAGGGTGC | R | CCTCAACAACCTTCCA |
| PGF | rs2268615 | ACGTTGGATGTCCTTTCCTCACCCCACATC | ACGTTGGATGGGGAAAGTGGCAGATTCCAG | F | caccTCCCACCATGCAGGTTC |
| TNFAIP2 | rs710100 | ACGTTGGATGATTGCAGGGCCAGCCTGTAG | ACGTTGGATGTTTTAGGGTCCTGTGGCGAG | F | ggCCTGGATGTGACGTCTAA |

SNP: single nucleotide polymorphism; UEP: unextended mini sequencing primer; DIR: direction; SEQ, sequence.

Supplementary **Table 3. The information and HWE about the candidate SNPs**

| **Gene** | **SNP ID** | **Chr:Position** | **Role** | **Alleles**  **(A/B)** | **MAF** | | ***p*-value for HWE** | **Call rate** | **OR (95%CI)** | ***p*** | **Haploreg** |
| --- | --- | --- | --- | --- | --- | --- | --- | --- | --- | --- | --- |
|  |  |  |  |  | **Cases** | **Controls** |  |  |  |  |  |
| PGF | rs8019391 | 14:74942979 | intronic | T/C | 0.22 | 0.20 | 0.067 | 100% | 1.13 (0.89-1.44) | 0.318 | Promoter histone marks, Enhancer histone marks, DNAse, Motifs changed, Selected eQTL hits |
| PGF | rs2268615 | 14:74951714 | intronic | A/C | 0.30 | 0.26 | 0.814 | 99.7% | **1.27 (1.03-1.58)** | **0.029** | Promoter histone marks, Enhancer histone marks, DNAse, Motifs changed, Selected eQTL hits |
| TNFAIP2 | rs710100 | 14:103135941 | 3′UTR | A/G | 0.40 | 0.36 | 0.200 | 99.1% | **1.23 (1.01-1.50)** | **0.043** | Enhancer histone marks, DNAse, Motifs changed, Selected eQTL hits |

HWE, Hardy-Weinberg equilibrium; SNP, single nucleotide polymorphism; MAF, minor allele frequency; eQTL, expression quantitative trait loci.
